# 2,3,3-Trimethyl-1-[4-(2,3,3-trimethyl-3*H*-indol-1-ium-1-yl)but­yl]-3*H*-indol-1-ium diiodide

**DOI:** 10.1107/S1600536812035234

**Published:** 2012-08-15

**Authors:** Di Wu, Li-Xia Shen, Yun-Yin Niu, Seik Weng Ng

**Affiliations:** a College of Chemistry and Molecular Engineering, Zhengzhou University, Zhengzhou, People’s Republic of China; b Department of Chemistry, University of Malaya, 50603 Kuala Lumpur, Malaysia; c Chemistry Department, King Abdulaziz University, PO Box 80203 Jeddah, Saudi Arabia

## Abstract

In the crystal of the title salt, C_26_H_34_N_2_^2+^·2I^−^, the dication lies on a center of inversion that exists along the mid-point of the butyl chain; its five-membered ring is approximately planar (r.m.s. deviation = 0.011 Å). In the crystal, the iodide anion is disordered over two positions in a 1:1 ratio.

## Related literature

For the synthesis, see: Yang *et al.* (2005 ▶). For industrial applications of Mannich products, see: Su *et al.* (2005 ▶).
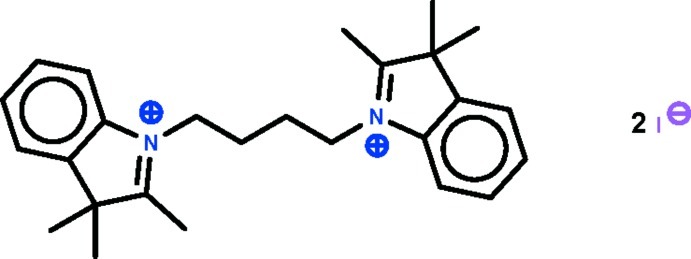


## Experimental

### 

#### Crystal data

C_26_H_34_N_2_^2+^·2I^−^
*M* *_r_* = 628.35
Monoclinic, 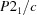

*a* = 13.9414 (14) Å
*b* = 7.6013 (8) Å
*c* = 13.8261 (15) Å
β = 113.011 (2)°
*V* = 1348.6 (2) Å^3^
*Z* = 2
Mo *K*α radiation
μ = 2.35 mm^−1^
*T* = 293 K
0.35 × 0.30 × 0.25 mm

#### Data collection

Bruker SMART APEX diffractometer
Absorption correction: multi-scan (*SADABS*; Sheldrick, 1996 ▶) *T*_min_ = 0.494, *T*_max_ = 0.592
8426 measured reflections
3077 independent reflections
2602 reflections with *I* > 2σ(*I*)
*R*_int_ = 0.017

#### Refinement

*R*[*F* ^2^ > 2σ(*F* ^2^)] = 0.027
*wR*(*F* ^2^) = 0.087
*S* = 1.04
3077 reflections
146 parameters
H-atom parameters constrained
Δρ_max_ = 0.94 e Å^−3^
Δρ_min_ = −0.37 e Å^−3^

### 

Data collection: *APEX2* (Bruker, 2005 ▶); cell refinement: *SAINT* (Bruker, 2005 ▶); data reduction: *SAINT*; program(s) used to solve structure: *SHELXS97* (Sheldrick, 2008 ▶); program(s) used to refine structure: *SHELXL97* (Sheldrick, 2008 ▶); molecular graphics: *X-SEED* (Barbour, 2001 ▶); software used to prepare material for publication: *publCIF* (Westrip, 2010 ▶).

## Supplementary Material

Crystal structure: contains datablock(s) global, I. DOI: 10.1107/S1600536812035234/xu5603sup1.cif

Structure factors: contains datablock(s) I. DOI: 10.1107/S1600536812035234/xu5603Isup2.hkl

Supplementary material file. DOI: 10.1107/S1600536812035234/xu5603Isup3.cml

Additional supplementary materials:  crystallographic information; 3D view; checkCIF report

## Figures and Tables

**Fig. 1.**
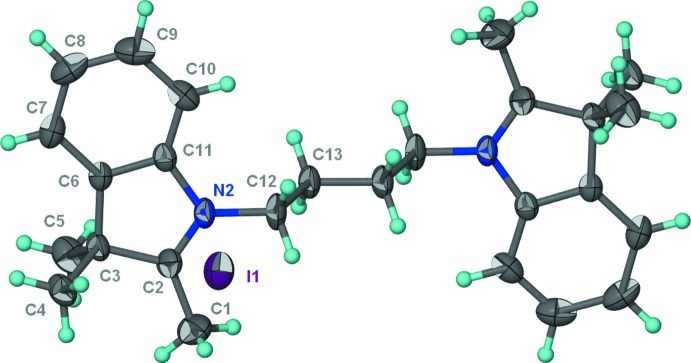
Thermal ellipsoid plot (Barbour, 2001) of C26H34N22+ 2I- at the 50% probability level; hydrogen atoms are drawn as spheres of arbitrary radius. The disorder in the iodine atom is not shown.

## supplementary crystallographic information

### Comment

The salt (Scheme I) can be synthesized in several steps from benzohydrazine, methyl isopropyl ketone and 1,4-diiodobutane, and is an intermediate in the synthesis of a bis-spironaphthoxazine (Yang *et al.*, 2005). It is available commercially in milligram quantities. The methyl group on the carbon atom adjacent to the quarternary nitrogen is acidic, and the methyl group undergoes a Mannich-type of reaction to yield compounds that have been patented for use as high-density recording media (Su *et al.*, 2005).

In the salt, C_26_H_34_N_2_^+^ 2I^-^, the dicationic species lies on a center-of-inversion that exists along the mid-point of the butyl chain; its five-membered ring is planar (r.m.s. deviation 0.011 Å). The iodide anion is disordered over two positions in a 1:1 ratio (Fig. 1).

### Experimental

3,3-Dimethyl-3*H*-indole (40 mmol, 5.81 g), 1,4-dibromobutane (20 mmol, 4.32 g) and potassium iodide (50 mmol, 8.310) were added to acetonitrile (40 ml). The mixture was heated under reflux for 4 days. The mixture was collected and the yellow solid recrystallized from a methanol-ether mixture; yield 40%. The salt is soluble in most organic solvents.

### Refinement

Carbon-, nitrogen- and oxygen-bound H-atoms were placed in calculated positions (C–H 0.93 to 0.97, N–H 0.88, O–H 0.84 Å) and were included in the refinement in the riding model approximation, with *U*(H) set to 1.2 to 1.5*U*(C,*N*,*O*).

The iodine atom is disordered over two positions; as the disorder refined to a nearly 1:1 ratio, the occupancy was then fixed as exactly 0.5.

### Crystal data

**Table:**

C_26_H_34_N_2_^2+^·2I^−^	*F*(000) = 620
*M**_r_* = 628.35	*D*_x_ = 1.547 Mg m^−^^3^
Monoclinic, *P*2_1_/*c*	Mo *K*α radiation, λ = 0.71073 Å
Hall symbol: -P 2ybc	Cell parameters from 3950 reflections
*a* = 13.9414 (14) Å	θ = 3.0–28.3°
*b* = 7.6013 (8) Å	µ = 2.35 mm^−^^1^
*c* = 13.8261 (15) Å	*T* = 293 K
β = 113.011 (2)°	Prism, colorless
*V* = 1348.6 (2) Å^3^	0.35 × 0.30 × 0.25 mm
*Z* = 2	

### Data collection

**Table:**

Bruker SMART APEX diffractometer	3077 independent reflections
Radiation source: fine-focus sealed tube	2602 reflections with *I* > 2σ(*I*)
Graphite monochromator	*R*_int_ = 0.017
ω scans	θ_max_ = 27.5°, θ_min_ = 3.0°
Absorption correction: multi-scan (*SADABS*; Sheldrick, 1996)	*h* = −13→17
*T*_min_ = 0.494, *T*_max_ = 0.592	*k* = −8→9
8426 measured reflections	*l* = −17→14

### Refinement

**Table:**

Refinement on *F*^2^	Primary atom site location: structure-invariant direct methods
Least-squares matrix: full	Secondary atom site location: difference Fourier map
*R*[*F*^2^ > 2σ(*F*^2^)] = 0.027	Hydrogen site location: inferred from neighbouring sites
*wR*(*F*^2^) = 0.087	H-atom parameters constrained
*S* = 1.04	*w* = 1/[σ^2^(*F*_o_^2^) + (0.0519*P*)^2^ + 0.3658*P*] where *P* = (*F*_o_^2^ + 2*F*_c_^2^)/3
3077 reflections	(Δ/σ)_max_ = 0.001
146 parameters	Δρ_max_ = 0.94 e Å^−^^3^
0 restraints	Δρ_min_ = −0.37 e Å^−^^3^

### Fractional atomic coordinates and isotropic or equivalent isotropic displacement parameters (Å^2^)

**Table:**

	*x*	*y*	*z*	*U*_iso_*/*U*_eq_	Occ. (<1)
I1	0.3111 (2)	0.6289 (3)	0.77695 (19)	0.0536 (2)	0.50
I1'	0.3220 (2)	0.6038 (3)	0.7930 (2)	0.0800 (6)	0.50
N2	0.31190 (14)	0.3641 (2)	0.55053 (14)	0.0382 (4)	
C1	0.3943 (2)	0.1407 (5)	0.6859 (3)	0.0789 (11)	
H1A	0.3772	0.0208	0.6943	0.118*	
H1B	0.4081	0.2039	0.7501	0.118*	
H1C	0.4550	0.1435	0.6693	0.118*	
C2	0.30596 (17)	0.2235 (3)	0.59984 (18)	0.0437 (5)	
C3	0.19566 (18)	0.1564 (3)	0.56180 (19)	0.0398 (5)	
C4	0.1613 (2)	0.1429 (3)	0.6543 (2)	0.0514 (6)	
H4A	0.1669	0.2562	0.6867	0.077*	
H4B	0.2051	0.0604	0.7049	0.077*	
H4C	0.0902	0.1035	0.6292	0.077*	
C5	0.1875 (3)	−0.0238 (4)	0.5086 (2)	0.0621 (7)	
H5A	0.2092	−0.0131	0.4510	0.093*	
H5B	0.1166	−0.0640	0.4830	0.093*	
H5C	0.2316	−0.1067	0.5586	0.093*	
C6	0.13926 (16)	0.2961 (3)	0.48332 (16)	0.0358 (4)	
C7	0.03506 (19)	0.3190 (3)	0.4208 (2)	0.0484 (5)	
H7	−0.0147	0.2402	0.4239	0.058*	
C8	0.0064 (2)	0.4603 (4)	0.3539 (2)	0.0585 (7)	
H8	−0.0635	0.4773	0.3112	0.070*	
C9	0.0803 (3)	0.5787 (4)	0.3488 (2)	0.0585 (7)	
H9	0.0590	0.6731	0.3025	0.070*	
C10	0.1846 (2)	0.5586 (3)	0.41111 (19)	0.0475 (5)	
H10	0.2345	0.6375	0.4084	0.057*	
C11	0.21086 (17)	0.4165 (3)	0.47728 (16)	0.0352 (4)	
C12	0.40689 (18)	0.4695 (4)	0.56978 (19)	0.0491 (6)	
H12	0.4593	0.4384	0.6380	0.059*	
H12B	0.3904	0.5931	0.5719	0.059*	
C13	0.45182 (18)	0.4435 (3)	0.48771 (18)	0.0435 (5)	
H13	0.4001	0.4747	0.4191	0.052*	
H13B	0.4699	0.3206	0.4858	0.052*	

### Atomic displacement parameters (Å^2^)

**Table:**

	*U*^11^	*U*^22^	*U*^33^	*U*^12^	*U*^13^	*U*^23^
I1	0.0481 (5)	0.0585 (3)	0.0626 (3)	−0.0023 (3)	0.0307 (3)	−0.0126 (3)
I1'	0.0531 (7)	0.0987 (14)	0.1030 (14)	−0.0154 (8)	0.0466 (9)	−0.0430 (8)
N2	0.0294 (9)	0.0511 (10)	0.0395 (9)	−0.0101 (7)	0.0193 (7)	−0.0044 (7)
C1	0.0413 (16)	0.107 (3)	0.083 (2)	0.0150 (15)	0.0190 (15)	0.0413 (18)
C2	0.0320 (11)	0.0575 (14)	0.0466 (12)	0.0011 (9)	0.0206 (9)	0.0075 (10)
C3	0.0342 (11)	0.0396 (11)	0.0514 (12)	−0.0026 (8)	0.0230 (9)	0.0066 (9)
C4	0.0516 (15)	0.0559 (14)	0.0590 (15)	−0.0019 (11)	0.0348 (12)	0.0082 (11)
C5	0.0748 (19)	0.0467 (14)	0.0763 (19)	0.0018 (13)	0.0420 (16)	−0.0007 (13)
C6	0.0317 (10)	0.0376 (10)	0.0406 (10)	−0.0031 (8)	0.0168 (8)	−0.0027 (8)
C7	0.0343 (12)	0.0543 (13)	0.0556 (14)	−0.0054 (10)	0.0166 (10)	−0.0083 (11)
C8	0.0460 (15)	0.0679 (17)	0.0498 (14)	0.0172 (13)	0.0058 (11)	−0.0049 (12)
C9	0.075 (2)	0.0531 (14)	0.0478 (14)	0.0189 (13)	0.0246 (13)	0.0090 (11)
C10	0.0623 (15)	0.0396 (11)	0.0493 (12)	−0.0011 (11)	0.0313 (11)	0.0025 (10)
C11	0.0354 (11)	0.0383 (10)	0.0365 (10)	−0.0052 (8)	0.0191 (8)	−0.0036 (8)
C12	0.0368 (12)	0.0683 (16)	0.0487 (12)	−0.0237 (11)	0.0237 (10)	−0.0136 (11)
C13	0.0343 (11)	0.0568 (13)	0.0459 (12)	−0.0135 (10)	0.0226 (9)	−0.0053 (10)

### Geometric parameters (Å, º)

**Table:**

N2—C2	1.287 (3)	C6—C11	1.381 (3)
N2—C11	1.433 (3)	C6—C7	1.381 (3)
N2—C12	1.480 (3)	C7—C8	1.371 (4)
C1—C2	1.478 (4)	C7—H7	0.9300
C1—H1A	0.9600	C8—C9	1.390 (4)
C1—H1B	0.9600	C8—H8	0.9300
C1—H1C	0.9600	C9—C10	1.378 (4)
C2—C3	1.507 (3)	C9—H9	0.9300
C3—C6	1.502 (3)	C10—C11	1.370 (3)
C3—C4	1.535 (3)	C10—H10	0.9300
C3—C5	1.538 (4)	C12—C13	1.508 (3)
C4—H4A	0.9600	C12—H12	0.9700
C4—H4B	0.9600	C12—H12B	0.9700
C4—H4C	0.9600	C13—C13^i^	1.517 (4)
C5—H5A	0.9600	C13—H13	0.9700
C5—H5B	0.9600	C13—H13B	0.9700
C5—H5C	0.9600		
			
C2—N2—C11	110.85 (18)	C11—C6—C7	119.1 (2)
C2—N2—C12	126.3 (2)	C11—C6—C3	109.00 (19)
C11—N2—C12	122.67 (19)	C7—C6—C3	131.9 (2)
C2—C1—H1A	109.5	C8—C7—C6	118.6 (2)
C2—C1—H1B	109.5	C8—C7—H7	120.7
H1A—C1—H1B	109.5	C6—C7—H7	120.7
C2—C1—H1C	109.5	C7—C8—C9	121.0 (2)
H1A—C1—H1C	109.5	C7—C8—H8	119.5
H1B—C1—H1C	109.5	C9—C8—H8	119.5
N2—C2—C1	125.0 (2)	C10—C9—C8	121.2 (2)
N2—C2—C3	111.39 (19)	C10—C9—H9	119.4
C1—C2—C3	123.5 (2)	C8—C9—H9	119.4
C6—C3—C2	100.79 (17)	C11—C10—C9	116.5 (2)
C6—C3—C4	113.8 (2)	C11—C10—H10	121.8
C2—C3—C4	109.9 (2)	C9—C10—H10	121.8
C6—C3—C5	111.4 (2)	C10—C11—C6	123.5 (2)
C2—C3—C5	110.1 (2)	C10—C11—N2	128.6 (2)
C4—C3—C5	110.5 (2)	C6—C11—N2	107.90 (18)
C3—C4—H4A	109.5	N2—C12—C13	113.52 (18)
C3—C4—H4B	109.5	N2—C12—H12	108.9
H4A—C4—H4B	109.5	C13—C12—H12	108.9
C3—C4—H4C	109.5	N2—C12—H12B	108.9
H4A—C4—H4C	109.5	C13—C12—H12B	108.9
H4B—C4—H4C	109.5	H12—C12—H12B	107.7
C3—C5—H5A	109.5	C12—C13—C13^i^	110.4 (2)
C3—C5—H5B	109.5	C12—C13—H13	109.6
H5A—C5—H5B	109.5	C13^i^—C13—H13	109.6
C3—C5—H5C	109.5	C12—C13—H13B	109.6
H5A—C5—H5C	109.5	C13^i^—C13—H13B	109.6
H5B—C5—H5C	109.5	H13—C13—H13B	108.1
			
C11—N2—C2—C1	−175.6 (3)	C3—C6—C7—C8	−178.7 (2)
C12—N2—C2—C1	−0.1 (4)	C6—C7—C8—C9	−0.1 (4)
C11—N2—C2—C3	2.1 (3)	C7—C8—C9—C10	−0.4 (4)
C12—N2—C2—C3	177.6 (2)	C8—C9—C10—C11	0.2 (4)
N2—C2—C3—C6	−2.7 (3)	C9—C10—C11—C6	0.3 (4)
C1—C2—C3—C6	175.0 (3)	C9—C10—C11—N2	−179.6 (2)
N2—C2—C3—C4	−123.1 (2)	C7—C6—C11—C10	−0.7 (3)
C1—C2—C3—C4	54.6 (3)	C3—C6—C11—C10	178.7 (2)
N2—C2—C3—C5	114.9 (2)	C7—C6—C11—N2	179.17 (19)
C1—C2—C3—C5	−67.3 (3)	C3—C6—C11—N2	−1.4 (2)
C2—C3—C6—C11	2.4 (2)	C2—N2—C11—C10	179.5 (2)
C4—C3—C6—C11	120.0 (2)	C12—N2—C11—C10	3.7 (3)
C5—C3—C6—C11	−114.4 (2)	C2—N2—C11—C6	−0.4 (3)
C2—C3—C6—C7	−178.3 (2)	C12—N2—C11—C6	−176.17 (19)
C4—C3—C6—C7	−60.7 (3)	C2—N2—C12—C13	104.5 (3)
C5—C3—C6—C7	65.0 (3)	C11—N2—C12—C13	−80.5 (3)
C11—C6—C7—C8	0.6 (4)	N2—C12—C13—C13^i^	179.6 (3)

Symmetry code: (i) −*x*+1, −*y*+1, −*z*+1.
